# Supplementary material for: Host plant phylogeny predicts arbuscular mycorrhizal fungal communities, but plant life history and fungal genetic change predict feedback
Source: PLoS Biol. 2026 Feb 25;24(2):e3003304. doi: 10.1371/journal.pbio.3003304 (PMC12962545; doi:10.1371/journal.pbio.3003304)
Supplement: S1 Fig — Plant species were selected to represent a phylogenetically diverse group of early and late successional plants. Plants were selected from 5 large phylogenetic groups; grasses (Poaceae), legumes (Fabaceae), asters (Asteraceae and Apiaceae), mints (Lamiaceae and Plantaginaceae), milkweeds (Apocynaceae and Asclepiadaceae), and lilies (Liliaceae and Commelinaceae). (DOCX) [file pbio.3003304.s001.docx]

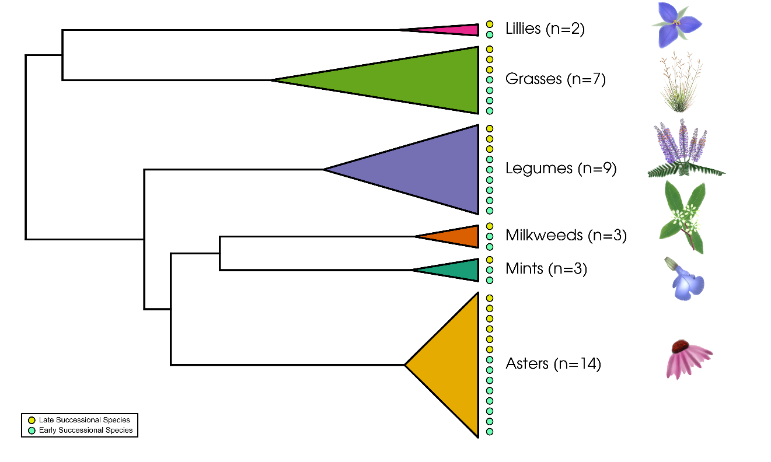


**S1 Fig. Host Plant Characteristics**
Plant species were selected to represent a phylogenetically diverse group of early and late successional plants. Plants were selected from 5 large phylogenetic groups; grasses (Poaceae), legumes (Fabaceae), asters (Asteraceae and Apiaceae), mints (Lamiaceae and Plantaginaceae), milkweeds (Apocynaceae and Asclepiadaceae), and lilies (Liliaceae and Commelinaceae).
