# Supplementary material for: Host plant phylogeny predicts arbuscular mycorrhizal fungal communities, but plant life history and fungal genetic change predict feedback
Source: PLoS Biol. 2026 Feb 25;24(2):e3003304. doi: 10.1371/journal.pbio.3003304 (PMC12962545; doi:10.1371/journal.pbio.3003304)
Supplement: S2 Fig — These are the estimated marginal means of AM fungal relative abundances when host plants were early or late successional. AM fungal species are arranged top to bottom in order of most to least beneficial. The data and code underlying this Figure can be found in https://doi.org/10.17605/OSF.IO/NAXMT. (DOCX) [file pbio.3003304.s002.docx]

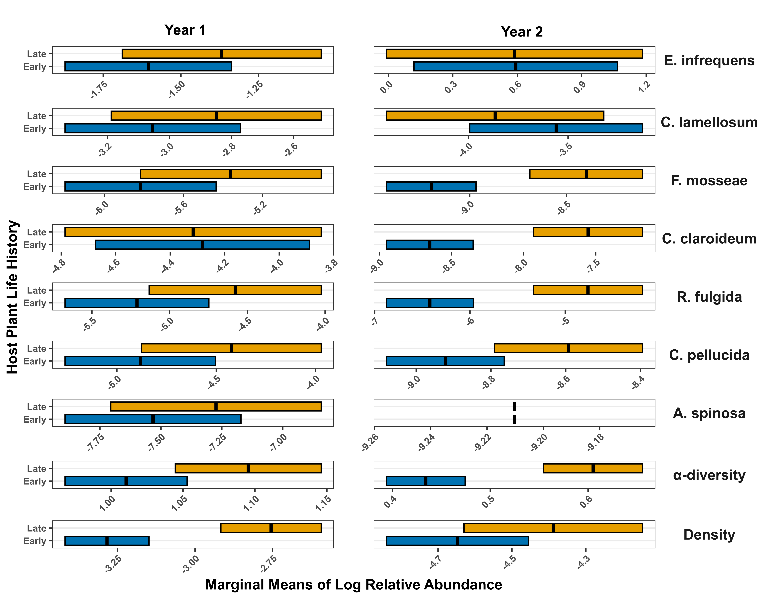


**S2 Fig. Marginal Means of Relative Abundance by Plant Life History**
These are the estimated marginal means of AM fungal relative abundances when host plants were early or late successional. AM fungal species are arranged top to bottom in order of most to least beneficial. The data and code underlying this Figure can be found in <https://doi.org/10.17605/OSF.IO/NAXMT>.
