# Supplementary material for: Host plant phylogeny predicts arbuscular mycorrhizal fungal communities, but plant life history and fungal genetic change predict feedback
Source: PLoS Biol. 2026 Feb 25;24(2):e3003304. doi: 10.1371/journal.pbio.3003304 (PMC12962545; doi:10.1371/journal.pbio.3003304)
Supplement: S3 Fig — The marginal means for the log response ratio’s (LRT) of each of the seven AM fungal species used in this study. The LRT is the natural log of the ratio between how large plant species grew with AM fungal inoculation versus how they grew in a sterile control. These are derived from a meta-analysis of three previous experiments using these same cultures. (A) shows late successional plant benefits when grown in association with E. infrequens, Cl. lamellosum, F. mosseae, and Cl. claroideum. (B) shows the marginal means for the log response ratio’s (LRT) of each of the seven AM fungal species used in this study by host plant phylogenetic group. Asteraceae experienced significantly greater growth than Poaceae in association with all Claroideoglomus species (including E. infrequens). We also saw significant differential greater growth in Liliaceae over Poaceae as well as Liliaceae over Asteraceae in several AM fungal species. The data and code underlying this Figure can be found in https://doi.org/10.17605/OSF.IO/NAXMT. (DOCX) [file pbio.3003304.s003.docx]

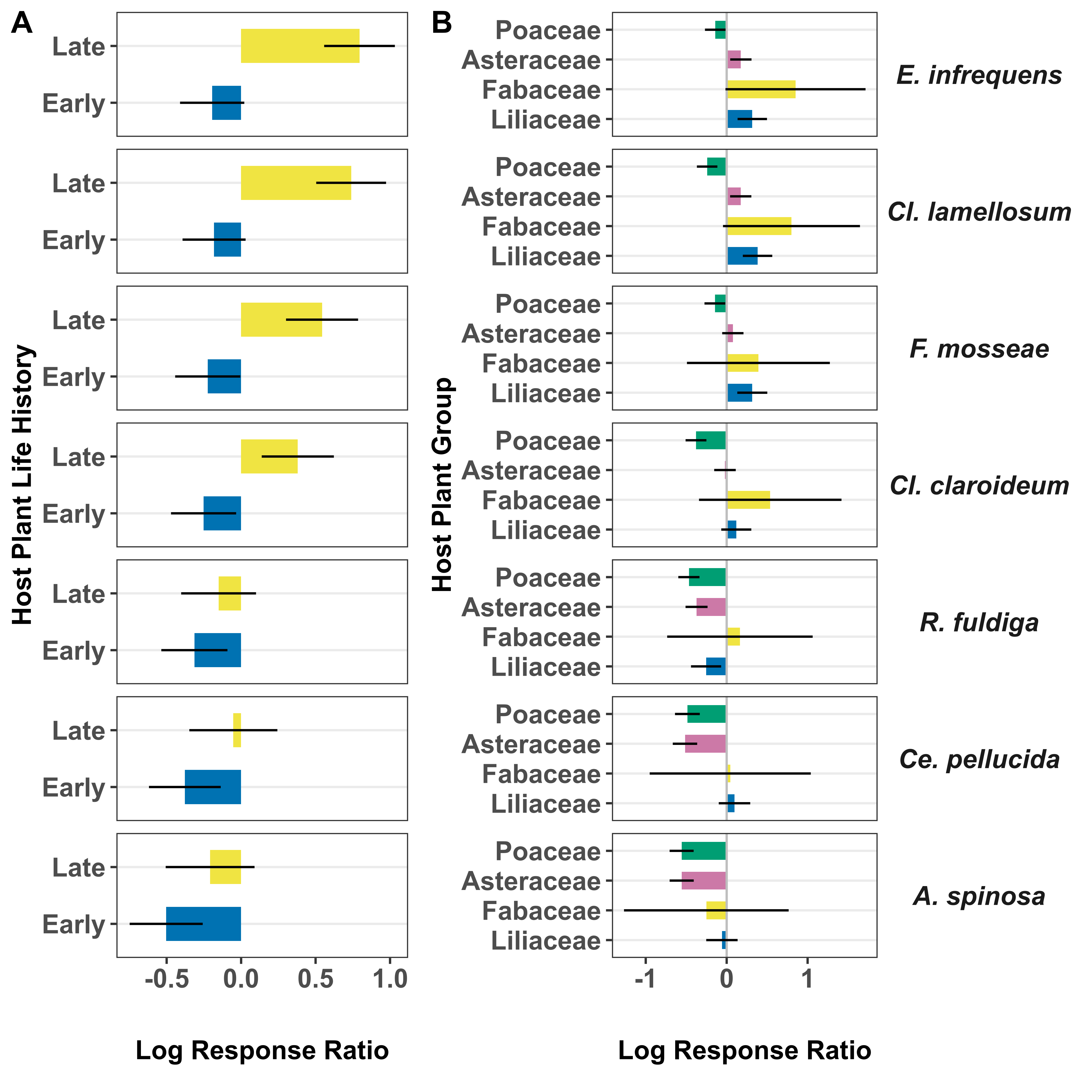


**S3 Fig. Meta-Analysis Results**
The marginal means for the log response ratio’s (LRT) of each of the seven AM fungal species used in this study. The LRT is the natural log of the ratio between how large plant species grew with AM fungal inoculation versus how they grew in a sterile control. The analysis containing both plant life history and plant phylogenetic group was significant (df = 34, QM = 163.3303, p-val < .0001). These are derived from a meta-analysis of three previous experiments using these same cultures. (A) shows late successional plant benefits when grown in association with *E. infrequens*, *Cl. lamellosum*, *F. mosseae*, and *Cl. claroideum*. The reduced model with only plant family was still significant (df = 13, QM = 90.8439, p-val < .0001). (B) shows the marginal means for the log response ratio’s (LRT) of each of the seven AM fungal species used in this study by host plant phylogenetic group. Asteraceae experienced significantly greater growth than Poaceae in association with all Claroideoglomus species (including *E. infrequens*). We also saw significant differential greater growth in Liliaceae over Poaceae as well as Liliaceae over Asteraceae in several AM fungal species. The reduced model containing only plant phylogenetic group was also significant (df = 27, QM = 69.3310, p-val < .0001). The data and code underlying this Figure can be found in <https://doi.org/10.17605/OSF.IO/NAXMT>.
