# Supplementary material for: Host plant phylogeny predicts arbuscular mycorrhizal fungal communities, but plant life history and fungal genetic change predict feedback
Source: PLoS Biol. 2026 Feb 25;24(2):e3003304. doi: 10.1371/journal.pbio.3003304 (PMC12962545; doi:10.1371/journal.pbio.3003304)
Supplement: S4 Fig — These are the log response ratios of the mean plant biomass for plants inoculated with a mixture of AM fungi to the mean plant biomass representative of the average single symbiont and to the most effective single symbiont respectively. Each panel breaks our results depending on if the host plant was an early or late successional. The data and code underlying this Figure can be found in https://doi.org/10.17605/OSF.IO/NAXMT. (DOCX) [file pbio.3003304.s004.docx]

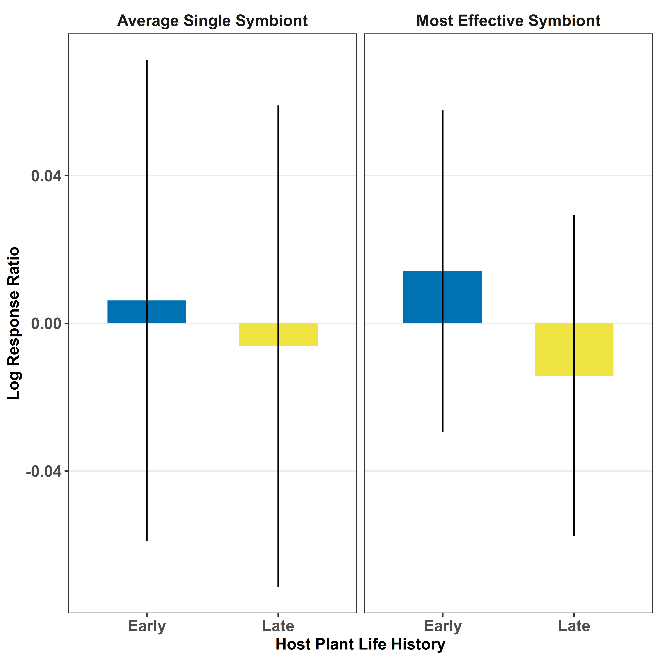


**S4 Fig. LRR by Life History of AM Fungal Mixtures to Single Symbiont** These are the log response ratios of the mean plant biomass for plants inoculated with a mixture of AM fungi to the mean plant biomass representative of the average single symbiont and to the most effective single symbiont respectively. Each panel breaks our results depending on if the host plant was an early or late successional. The data and code underlying this Figure can be found in <https://doi.org/10.17605/OSF.IO/NAXMT>.
