# Supplementary material for: Host plant phylogeny predicts arbuscular mycorrhizal fungal communities, but plant life history and fungal genetic change predict feedback
Source: PLoS Biol. 2026 Feb 25;24(2):e3003304. doi: 10.1371/journal.pbio.3003304 (PMC12962545; doi:10.1371/journal.pbio.3003304)
Supplement: S5 Fig — This shows counts of all paired comparisons with both the median and mean being significantly positive (p = 0.003, p ≤ 0.001). The data and code underlying this Figure can be found in https://doi.org/10.17605/OSF.IO/NAXMT. (DOCX) [file pbio.3003304.s005.docx]

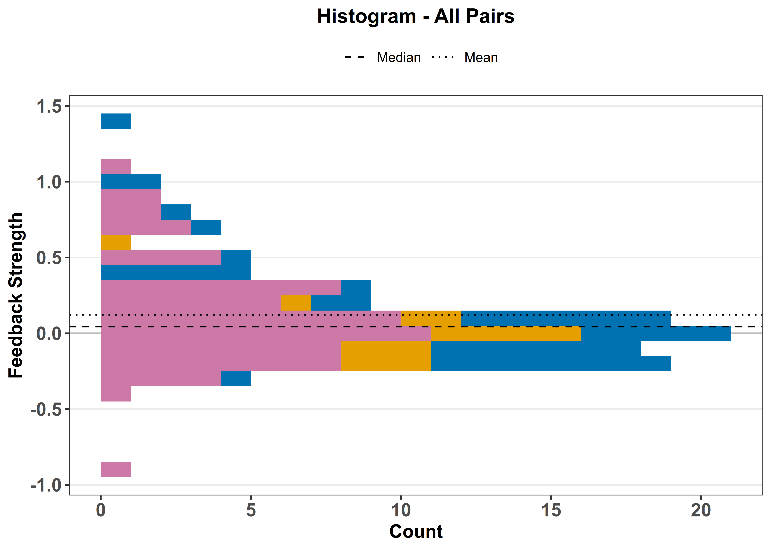


**S5 Fig. Histogram of mycorrhizal feedback measured as pairwise interaction coefficients**This shows counts of all paired comparisons with both the median and mean being significantly positive (p = 0.003, p≤ 0.001). The data and code underlying this Figure can be found in <https://doi.org/10.17605/OSF.IO/NAXMT>.
