# Supplementary material for: Host plant phylogeny predicts arbuscular mycorrhizal fungal communities, but plant life history and fungal genetic change predict feedback
Source: PLoS Biol. 2026 Feb 25;24(2):e3003304. doi: 10.1371/journal.pbio.3003304 (PMC12962545; doi:10.1371/journal.pbio.3003304)
Supplement: S6 Fig — Mycorrhizal feedback, as measured by the pairwise interaction coefficient, compared across phylogenetic group pairings. Feedback between individual pairs of plant species are indicated with dots, with pairwise feedback that was significantly different from zero indicated in black. We note that pairwise mycorrhizal feedbacks ranged from significantly negative to significantly positive. On average, we observed significantly positive mycorrhizal feedback between two legume species (p ≤ 0.01) and significant negative mycorrhizal feedback between aster and grass species (p ≤ 0.05). The data and code underlying this Figure can be found in https://doi.org/10.17605/OSF.IO/NAXMT. (DOCX) [file pbio.3003304.s006.docx]

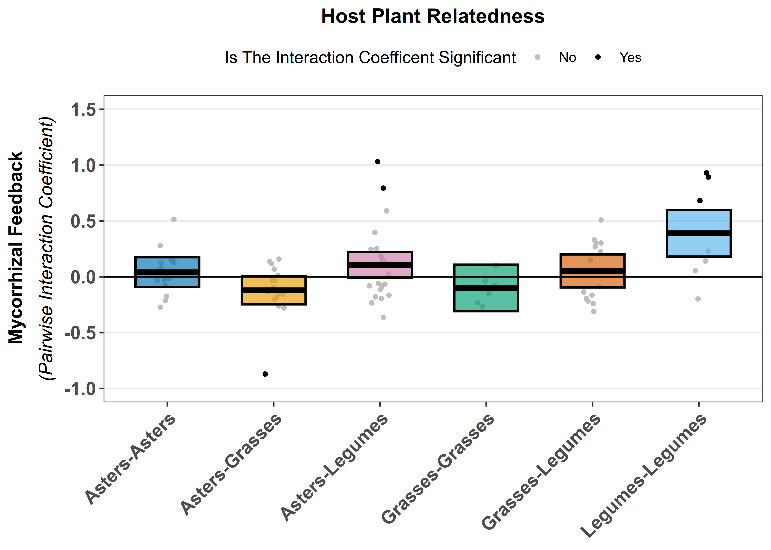

**S6 Fig. Feedbacks by Plant Phylogenetic Group** Mycorrhizal feedback, as measured by the pairwise interaction coefficient, compared across phylogenetic group pairings. Feedback between individual pairs of plant species are indicated with dots, with pairwise feedback that was significantly different from zero indicated in black. We note that pairwise mycorrhizal feedbacks ranged from significantly negative to significantly positive. On average, we observed significantly positive mycorrhizal feedback between two legume species (p≤0.01) and significant negative mycorrhizal feedback between aster and grass species (p≤0.05). The data and code underlying this Figure can be found in <https://doi.org/10.17605/OSF.IO/NAXMT>.
