# Supplementary material for: Host plant phylogeny predicts arbuscular mycorrhizal fungal communities, but plant life history and fungal genetic change predict feedback
Source: PLoS Biol. 2026 Feb 25;24(2):e3003304. doi: 10.1371/journal.pbio.3003304 (PMC12962545; doi:10.1371/journal.pbio.3003304)
Supplement: S7 Fig — Regressions of strength of pairwise feedback against measures of AM fungal genetic dissimilarity. Pairwise feedback was significantly predicted by genetic dissimilarity of two AM fungal species, E. infrequens (p < 0.001) and Cl. lamellosum (p < 0.001). The data and code underlying this Figure can be found in https://doi.org/10.17605/OSF.IO/NAXMT. (DOCX) [file pbio.3003304.s007.docx]

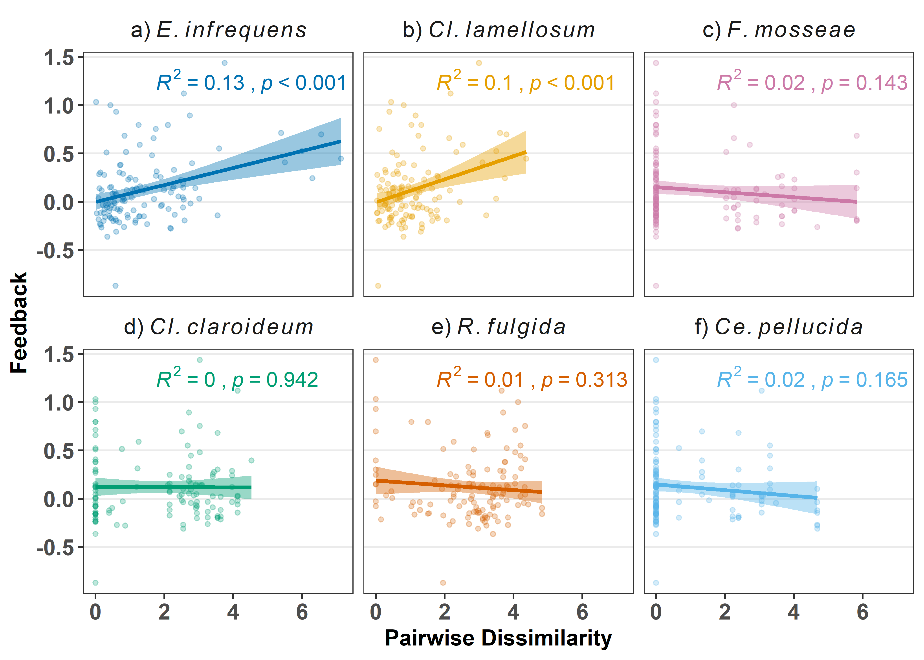


**S7 Fig. Mycorrhizal Feedback Strength Predicted by Genetic Dissimilarity of AM fungi for All AM fungal Species**Regressions of strength of pairwise feedback against measures of AM fungal genetic dissimilarity. Pairwise feedback was significantly predicted by genetic dissimilarity of two AM fungal species, *E. infrequens* (p<0.001, F=17.78=, R^2^=0.13) and *Cl. lamellosum* (p<0.001, F=13.27, R^2^=0.1). Other species were not significant, including *F. mosseae* (p=0.143, F=2.175, R^2^=0.02), *C. claroideum* (p=0.942, F=0.005, R^2^=0), *R. fulgida* (p=0.313, F=1.026, R^2^=0.01), and *Ce. pellucida* (p=0.165, F=1.952, R^2^=0.02). The data and code underlying this Figure can be found in <https://doi.org/10.17605/OSF.IO/NAXMT>.
