# Supplementary material for: Host plant phylogeny predicts arbuscular mycorrhizal fungal communities, but plant life history and fungal genetic change predict feedback
Source: PLoS Biol. 2026 Feb 25;24(2):e3003304. doi: 10.1371/journal.pbio.3003304 (PMC12962545; doi:10.1371/journal.pbio.3003304)
Supplement: S8 Fig — The proxy for AM fungal density is the proportion of inoculated AM fungal ASVs and sequencing depth. This was used because spore counts were not completed for all 156 training pots. The relationship between our proxy and spore count for those pots where data was available was statistically significant (p < 0.001). The data and code underlying this Figure can be found in https://doi.org/10.17605/OSF.IO/NAXMT. (DOCX) [file pbio.3003304.s008.docx]

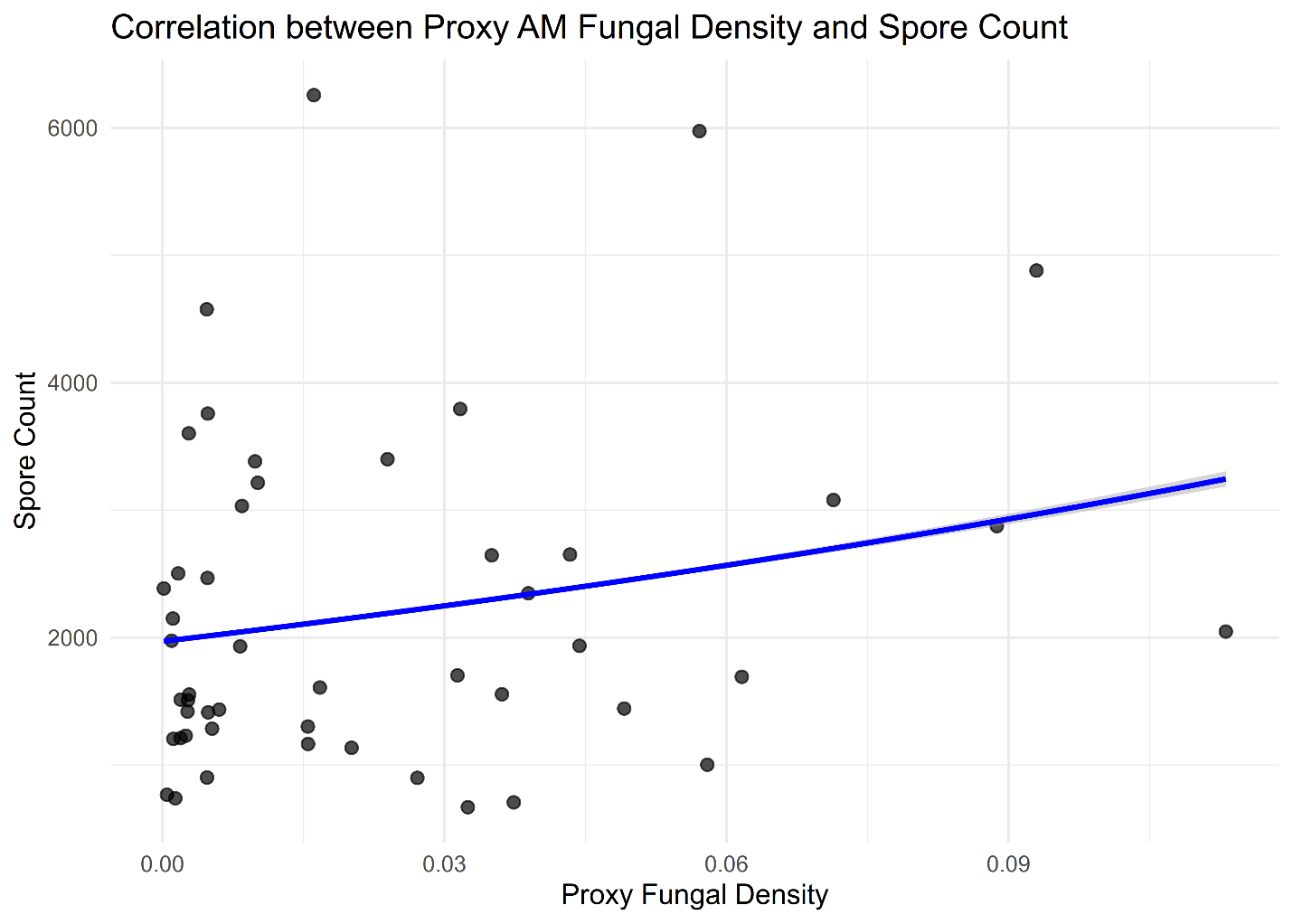


**S8 Fig. Correlation between Proxy AM fungal Density and Spore Count**
The proxy for AM fungal density is the proportion of inoculated AM fungal ASVs and sequencing depth. This was used because spore counts were not completed for all 312 training pots. The relationship between our proxy and spore count for those pots where data was available was statistically significant using a Poisson generalized linear model (p<0.001, McFadden Pseudo R2 = 0.051). The blue line represents the best fit line. The data and code underlying this Figure can be found in <https://doi.org/10.17605/OSF.IO/NAXMT>.
