# Supplementary material for: Host plant phylogeny predicts arbuscular mycorrhizal fungal communities, but plant life history and fungal genetic change predict feedback
Source: PLoS Biol. 2026 Feb 25;24(2):e3003304. doi: 10.1371/journal.pbio.3003304 (PMC12962545; doi:10.1371/journal.pbio.3003304)
Supplement: S9 Fig — The experimental design for the feedback experiment included 5 subset groups of plants where all plant pairings were made in a fully factorial design. Each group also included early and late successional plants. When these pairings are arranged phylogenetically it becomes clearer that we also have a good representation of species pairs across the plant phylogeny. This allows us to test pairwise feedbacks, host plant characteristics, and plant phylogeny effects in a single experiment. The data and code underlying this Figure can be found in https://doi.org/10.17605/OSF.IO/NAXMT. (DOCX) [file pbio.3003304.s009.docx]

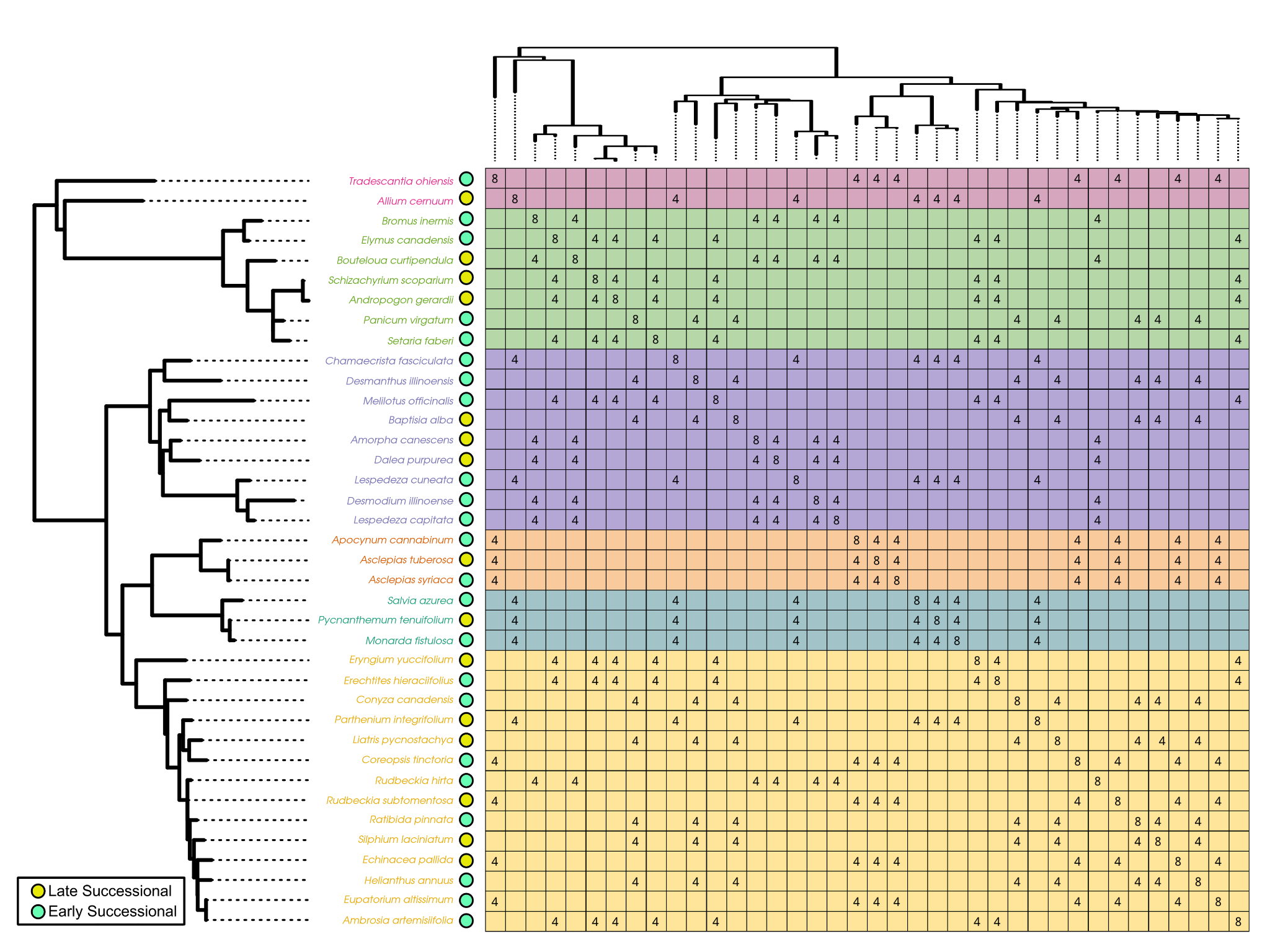


**S9 Fig. Feedback Experimental Design**

The experimental design for the feedback experiment included 5 subset groups of plants where all plant pairings were made in a fully factorial design. Each group also included early and late successional plants. When these pairings are arranged phylogenetically it becomes clearer that we also have a good representation of species pairs across the plant phylogeny. This allows us to test pairwise feedbacks, host plant characteristics, and plant phylogeny effects in a single experiment. The data and code underlying this Figure can be found in <https://doi.org/10.17605/OSF.IO/NAXMT>.
