# Supplementary material for: Host plant phylogeny predicts arbuscular mycorrhizal fungal communities, but plant life history and fungal genetic change predict feedback
Source: PLoS Biol. 2026 Feb 25;24(2):e3003304. doi: 10.1371/journal.pbio.3003304 (PMC12962545; doi:10.1371/journal.pbio.3003304)
Supplement: S2 Table — To test for overall differences in AM fungal composition with plant life history and plant family, we conducted a PerMANOVA on centered log ratio (CLR) transformed counts of ASVs grouped by AM fungal species. This analysis reveals statistically significant effects on AM fungal species composition of plant phylogenetic group in both years (p ≤ 0.001, p ≤ 0.001). We also tested for overall differences in AM fungal composition with plant life history and plant family using the PerMANOVA approach. This analysis reveals statistically significant effects on AM fungal species composition of plant life history on AM fungal species composition in year two (p < 0.001). (DOCX) [file pbio.3003304.s011.docx]

| **S2 Table. PerMANOVA Results for AM Fungi Species** | | | | | | | | |  |
| --- | --- | --- | --- | --- | --- | --- | --- | --- | --- |
|  | Year 1 | | | |  | Year 2 | | | |
|  | Df | R2 | Pr(>F) |  |  | Df | R2 | Pr(>F) |  |
| Plant life history | 1 | 0 | 1 |  |  | 1 | 0.03 | 0 | ٭٭٭ |
| Plant group | 5 | 0.1 | 0 | ٭٭٭ |  | 5 | 0.11 | 0 | ٭٭٭ |
| Plant species | 31 | 0.28 | 0 | ٭٭٭ |  | 30 | 0.29 | 0 | ٭٭٭ |
| Seq depth | 1 | 0 | 0.83 |  |  | 1 | 0.01 | 0.05 | ٭ |
| Block | 3 | 0.02 | 0.18 |  |  | 3 | 0.01 | 0.75 |  |
| Residual | 106 | 0.59 |  |  |  | 104 | 0.54 |  |  |
| Total | 147 | 1 |  |  |  | 144 | 1 |  |  |
| *** p ≤ 0.001; ** p ≤ 0.01; * p ≤ 0.05; · p ≤ 0.1 | | | | | | | | | |

To test for overall differences in AM fungal composition with plant life history and plant family, we conducted a PerMANOVA on centered log ratio (CLR) transformed counts of ASVs grouped by AM fungal species. This analysis reveals statistically significant effects on AM fungal species composition of plant phylogenetic group in both years (p≤0.001, p≤0.001). We also tested for overall differences in AM fungal composition with plant life history and plant family using the PerMANOVA approach. This analysis reveals statistically significant effects on AM fungal species composition of plant life history on AM fungal species composition in year two (p<0.001**)**
