# Supplementary material for: Host plant phylogeny predicts arbuscular mycorrhizal fungal communities, but plant life history and fungal genetic change predict feedback
Source: PLoS Biol. 2026 Feb 25;24(2):e3003304. doi: 10.1371/journal.pbio.3003304 (PMC12962545; doi:10.1371/journal.pbio.3003304)
Supplement: S4 Table — Phylogenetic generalized linear mixed model (PGLMM) results for relative proportions of each AM fungal species (combined ASV counts), as well as Shannon Diversity and Logit transformed density estimates. (DOCX) [file pbio.3003304.s013.docx]

| **S4 Table. PGLMM Results for AM Fungi Species** | | | | | | | | |
| --- | --- | --- | --- | --- | --- | --- | --- | --- |
|  |  | **R2** | **Host Life History** | **Host Species** | **Host Phylogentic** | **Block** | **Log Seq Depth** | **Residual** |
| *E. infrequens* | Year1 | 0.34 | 0.08 (0.21) | 0 (0) *** | 1.02 (0.6) *** | 0 (0) | 0 (0) | 2.84 (1.69) |
|  | Year2 | 0.20 | 0.05 (0.39) | 0.8 (1.83) *** | 1.63 (2.61) * | 0 (0) | 11.47 (6.93) | 0.24 (0.49) |
| *C. lammellosum* | Year1 | 0.15 | -0.04 (0.25) | 0.66 (0.38) *** | 0.37 (0.28) | 0.37 (0.28) * | 0 (0.02) | 4.57 (2.14) |
|  | Year2 | 0.07 | 0.05 (0.34) | 1.42 (2.58) | 0 (0.01) | 0.28 (1.15) | 11.17 (7.23) | 0.21 (0.46) |
| *F. mosseae* | Year1 | 0.06 | -0.18 (0.27) | 0 (0) | 0.04 (0.07) | 0 (0) | 2.38 (0.56) | 7.54 (2.75) |
|  | Year2 | 0.40 | -0.48 (0.28) . | 2.46 (4.1) *** | 0 (0) | 0 (0) | 1.5 (3.2) | 0.15 (0.38) |
| *C. claroidium* | Year1 | 0.09 | 0.12 (0.29) | 0 (0.01) | 0.28 (2.99) | 0 (0.03) | 10.73 (18.44) . | 0.03 (0.18) |
|  | Year2 | 0.10 | -0.54 (0.24) * | 0.58 (1.4) | 0.02 (0.28) | 0.04 (0.39) | 5.27 (4.23) | 0.29 (0.54) |
| *R. fulgida* | Year1 | 0.19 | -0.3 (0.45) | 4.43 (3.4) *** | 0.21 (0.74) | 0.4 (1.02) | 9.93 (5.09) | 0.38 (0.62) |
|  | Year2 | 0.26 | -0.68 (0.36) . | 0.6 (1.73) *** | 0.87 (2.09) *** | 0 (0) | 11.78 (7.67) | 0.2 (0.45) |
| *S. pellucida* | Year1 | 0.17 | -0.17 (0.3) | 0.03 (0.06) *** | 0.79 (0.3) * | 0.8 (0.3) * | 0 (0) | 8.91 (2.98) |
|  | Year2 | 0.10 | -0.18 (0.14) | 0.35 (1) ** | 0 (0) | 0 (0) | 0.99 (1.69) | 0.35 (0.59) |
| *A. spinosa* | Year1 | 0.08 | -0.09 (0.26) | 0.32 (317.43) | 0 (1.83) | 0 (7.41) | 8.17 (1597.02) | 0 (0) |
| *Shannon Diversity* | Year1 | 0.10 | -0.02 (0.03) | 0 (0) *** | 0.01 (0.55) * | 0 (0.07) | 0.07 (1.26) | 0.04 (0.21) |
|  | Year2 | 0.17 | -0.09 (0.04) * | 0.03 (0.71) *** | 0 (0) | 0 (0.26) . | 0.01 (0.36) | 0.07 (0.26) |
| *Logit*  *Density* | Year1 | 0.42 | -0.16 (0.11) | 0 (0.01) *** | 0.34 (3.05) *** | 0.01 (0.6) | 0.68 (4.31) | 0.04 (0.19) |
|  | Year2 | 0.25 | -0.02 (0.21) | 1.28 (1.57) *** | 0 (0) | 0.19 (0.6) ** | 1.04 (1.41) | 0.52 (0.72) |
| *** p<0.001; ** p<0.01; * p<0.05; · p<0.1 | | | | | | | | |

Phylogenetic generalized linear mixed model (PGLMM) results for relative proportions of each AM fungal species (combined ASV counts), as well as Shannon Diversity and Logit transformed density estimates.­
