# Supplementary material for: Host plant phylogeny predicts arbuscular mycorrhizal fungal communities, but plant life history and fungal genetic change predict feedback
Source: PLoS Biol. 2026 Feb 25;24(2):e3003304. doi: 10.1371/journal.pbio.3003304 (PMC12962545; doi:10.1371/journal.pbio.3003304)
Supplement: S5 Table — Phylogenetic heritability of microbiome composition describes the differential influence of plant species on microbiome composition as predicted by the plant phylogeny. A substantial proportion of variation, ranging from 0.000% for Cetraspora pellucida to 99.999% for Entrophosphora infrequens, in species impacts on microbiome composition that can be explained by plant phylogeny. (DOCX) [file pbio.3003304.s014.docx]

| **S5 Table. Phylogenetic heritability** | | | |
| --- | --- | --- | --- |
| **Group** | **Year** | **Phylogenetic heritability** |  |
| *Entrophosphora*  *infrequens* | **1** | 99.9% | *** |
|  | **2** | 67.0% | * |
| *Clariodeoglomus*  *lamellosum* | **1** | 35.5% |  |
|  | **2** | 0.001% |  |
| *Funneliformis mosseae* | **1** | 99.9% |  |
|  | **2** | 0.000% |  |
| *Clariodeoglomus*  *claroideum* | **1** | 99.9% |  |
|  | **2** | 3.81% |  |
| *Racocetra fulgida* | **1** | 4.57% |  |
|  | **2** | 59.3% | *** |
| *Cetraspora pellucida* | **1** | 96.1% | * |
|  | **2** | 0.000% |  |
| *Acaulospora spinosa* | **1** | 0.003% |  |
| *** p < 0.001; ** p < 0.01; * p < 0.05; · p < 0.1 | | | |

Previous work showing that microbiome composition can be influenced by plant genotype used measures of “microbiome heritability'' to represent the proportion of microbiome variation explained by plant genetics. We use an analogous metric of phylogenetic heritability of microbiome composition to describe the differential influence of plant species on microbiome composition as predicted by the plant phylogeny. These measures of “phylogenetic microbiome heritability” identify that a substantial proportion of variation, ranging from 0.000% for *Cetraspora pellucida* to 99.9% for *Entrophosphora infrequens*, in species impacts on microbiome composition that can be explained by plant phylogeny. We note that this influence on AM fungal composition does not appear to result from differential exclusion of AM fungal species from roots, as would be expected from incompatibilities of plant-AMF signaling, as inoculated AMF were found with all plant species. Rather, given that inoculated AMF had equivalent density at the beginning of the experiment, the change in composition is due to host-specific differences in AM fungal growth rates (i.e., fitness). This measure of microbiome heritability benefits from the ability of PGLMMs to distinguish the effects of ecological drivers from the underlying phylosymbiotic signal.
