# Supplementary material for: Host plant phylogeny predicts arbuscular mycorrhizal fungal communities, but plant life history and fungal genetic change predict feedback
Source: PLoS Biol. 2026 Feb 25;24(2):e3003304. doi: 10.1371/journal.pbio.3003304 (PMC12962545; doi:10.1371/journal.pbio.3003304)
Supplement: Attachment — Submitted filename: Response to Reviewers_11_28_2025.docx [file pbio.3003304.s015.docx]

REVIEWERS' COMMENTS:

Reviewer #1: PBIOLOGY-D-25-02002R1

Here the authors present a manuscript investigating plant host relatedness and AM fungal diversity and composition using an experimental setup of many grassland and fungal species. This research builds onto existing work on host-symbiont relations, and is novel in that the author's investigate mycorrhizal feedbacks onto host plants, over different years. Overall I find the research rigorous, however a few notes could improve the manuscript to fit well with the broad readership of PLOS. The main concern I have is digesting so many different methods into one clear story.

First, the manuscript can be considerably shortened, for example in the introduction covers many other symbioses than plant-AM fungal, but the results here are largely discussed in context of plants and AM fungi. It is a lot of work reading manuscripts so making it as clear and easy for the reader is a major goal of convincing them to continue onto the next paragraph of the paper. In this vain it would help to maybe have an introduction where it is structured in 1-2 paragraphs of the main problem and walk the reader through the different ways you test this, and why you test is in so many ways (as show in in Fig 1.)

**Response: We appreciate your desire for a streamlined introduction. We have done this within the goal of highlighting relevance of our work to multiple audiences and multiple ongoing intellectual conversations in ecology and evolution and addressing concerns of other reviewers. Specifically, we have collapsed what was previously 2 paragraphs (2 and 3) into one. And tightened all paragraphs.**

Likewise the use of a meta-analysis is welcome because it builds on previous research but no information is given on the power/confidence in these results.

**Response: We report the results of statistical analyses from the meta-analysis which guide the confidence in differential effects of these isolates on growth early and late successional prairie plant species and on growth of plants of different families. We therefore interpret the question as how much power do we have to test the pattern of relative growth rates of these plant groupings and the relative growth rates of the AM fungal isolates on these plant groupings, which will characterize the feedback (see Bever 1999 Ecology Letters). While working with 38 plant species generally gives us very high power to test patterns that we discuss (e.g. patterns of differentiation of AMF across phylogeny and across successional status), the power in tests of correlations of differential impacts and the differential responses of the AMF isolates is constrained by our inclusion of seven isolates. I.e. this test has lower power. However, our direct test of mycorrhizal feedback has high power, as it was independently assessed for 126 pairs of plant species. We now identify this difference in power within our discussion of these results.**

Lastly, the use of complex phylogenetic models is an advancement, but they also require more careful testing of your hypotheses, of which I suggest an addition to explain variance by phylogeny vs species effects uncorrelated to the host phylogeny, see reference in Ecol Letters below.

**Response: We have compiled a measure of phylogenetic heritability, (the proportion of the variance explained by phylogenetic relationships compared to the total variance explained by both species identity and phylogenetic effects. It was omitted in the service of streamlining the manuscript but has been added back in as supplement 13 for completeness.**

Due to the high number of experiments and analyses, it would benefit the text to use as little jargon as possible (e.g. abbreviations, I count at least 7 excluding the species names), different statistical tests names, plant-fungal species names). For example I see growth rates and fitness used through the manuscript (is this LRR?), please clearly define all terms especially if they are used to make claims in the discussion.

**Response: We agree with you. While the use of some jargon is sometime unavoidable with certain topics, we have aimed to minimize its use. We do appreciate the comment that there were a number of places we did not define our acronyms. We have gone through the manuscript and added clarifications where needed.**

I also suggest using active or passive voice consistently in the document.

**Response: We have attempted to use active voice where possible.**

Specific Comments:

46: I would define symbiont, since this is in review in a general journal

**Response: We have added and then deleted the definition to meet the editors direction to shorten the introduction.**

59: The jump here is quite quick from phylosymbioses exist to these phylosiybioses are important for fitness. When symbiosis could also be commensal and not influence fitness. A sentence on why the host may influence fitness and "composition", which I assume is community composition is needed. Guiding the reader through the logic will put less strain on the reader to understand why they should even care about testing for influences on fitness/competition. This sentence may actually do well to start the next paragraph.

**Response: We have combined these two paragraphs and added linkages of composition to host fitness.**

104: I am very happy to see more complex phylogenetic models being employed here.

**Response: Quite a lot of thought was put into the proper analysis framework for understanding the phylogenetic patterns. The comment is very appreciated.**

140: Why include the PERMANOVA at all, if you know the other model is better equipped to answer your question?

**Response: PERMANOVA controls for non-independence of multivariate responses while univariate tests control and test for phylogenetic non-independence. Together, we confirm that our results are robust to both forms of non-independence.**

142: Is a benefit of your mixed effect model being overlooked? If I understand your model structure correctly you can compare variance explained by phylogeny vs species identity here by looking at intraclass correlation coefficients. The ability to compare variance explained by phylogenetic relatedness of hosts vs species effects uncorrelated to host phylogeny would be welcome. Following: <https://nam10.safelinks.protection.outlook.com/?url=https%3A%2F%2Fonlinelibrary.wiley.com%2Fdoi%2Ffull%2F10.1111%2Fele.13263&data=05%7C02%7CRob.Ramos%40colorado.edu%7C68a79ed505304fd254ba08ddf9ef7ae7%7C3ded8b1b070d462982e4c0b019f46057%7C1%7C0%7C638941529135437743%7CUnknown%7CTWFpbGZsb3d8eyJFbXB0eU1hcGkiOnRydWUsIlYiOiIwLjAuMDAwMCIsIlAiOiJXaW4zMiIsIkFOIjoiTWFpbCIsIldUIjoyfQ%3D%3D%7C0%7C%7C%7C&sdata=kQXUVt%2BnonhPGQ1oQxtowSw7MjHX1tqoxk%2Fz0D%2F4mQ8%3D&reserved=0> .

**Response: Thank you for this comment. As mentioned above, we integrate representation of the relative magnitude of variation explained by phylogeny using a measure of phylogenetic heritability, which is based on the cross-product as the paper describes. We included it as supplement S7 Table.**

144: What is density? Of hyphae, biomass, spores?

**Response: Our measure of density is only a proxy. We did not have qPCR nor complete counts of spores to compute a more direct measure. It is simply the proportion of our inoculated species ASVs over the total sequencing depth. We recognize this is a very imperfect measure, and while a discussion of this metric was present, we will expand it to better highlight the limitations. We do find that this measure is positively correlated with the counts of spores in the subsample of pots for which we have this measure. We now include this result in supplement S13 Table.**

Figure 2: Define the color bar in the caption please.

**Response: I have added two sentences further explaining our relative proportion metric. I have also updated the figure legend to now read “Relative Proportion” instead of just proportion.**

165-166 "third most abundant ASV had a significant effect of host plant phylogenetic distance in year 1 (p<0.01, p<0.001, p<0.001)."

**Response. We thank the reviewer for pointing out this discrepancy. There were a few results in this paragraph that were mistakenly referring to p-values from an older analysis. We have corrected this error.**

179: How does one measure marginally significant higher? Or do you have greater confidence in sig based on the p-value?
Maybe I missed it but here and in other areas breaking this sentence down what does it mean if an ASV has an effect on host phylo distance. I would consider if effect is the correct word choice, because it is not clear what an ASV effect on phlyo distance would be.

**Response: We only refer to statistical confidence. We agree that the current wording might be confusing. We have adjusted the paragraph to more clearly state that we observe a correlation between host plant life history category and the relative proportion of certain ASVs with marginal statistical confidence. The author’s also don’t posit that AMF ASV relative abundance has any effect on host plant phylogenetic relationships, but rather that the phylogenetic relationships could show closely related host plants having similar effects on AMF fitness. I have attempted to clarify this point in the figure caption.**

Discussion General: It is clear much work has been done in this manuscript, and to help the reader it would be good to refer to the figures with your main discussion points. There is quite a lot of information, tests, and analyses here so really walking the reader through and in the discussion pointing back to specific parts of the results is welcome. This occurs once for Figure 2, but it is helpful to do more throughout. This is especially helpful for discussion paragraph one where you recap all the work done and distil it for the reader.

**Response: We have attempted to clarify the figure legends and results section to better walk through the findings.**

Further, here it seems that mycorrhizal feedbacks may also be time dependent. If fungal and host phenology are aligned, then would you expect ± correlations. Given that AM fungi grow from carbon that has to be captured by the plant host and then traded to the fungi, it would be expected that hyphae and spores grow lagged compared to high plant productivity. It would be worth discussing the effects of time in a brief 1-2 sentences, and if the time of your sampling may bias measurements of feedback. E.g. line 339.

**Response: We included in the methods a brief discussion about time. We sampled at the end of growing season and do not believe that any lag in response to carbon acquisition would be detectable at the time of plant senescence. We note that you are thinking of physiological feedbacks. Our goal is to assess feedbacks due to change in mycorrhizal composition. We have timed the study so that the AM fungi produce spores at the end of the growing season, and then the samples are stored for a period of time to break dormancy and then their growth impact is assessed. Our final analysis suggests that the change in genetic composition that we measured in two AM fungal species is the major driver of the feedbacks over this time frame.**

211: Here and in other places remind the reader what you are doing at each test, since this is so many things in one manuscrtip. "Next we conducted a meta-analysis of XXX to answer YYY". Our results show ZZZ.

**Response: We have edited the results in several locations to clearly state the analysis, question, and overall result where it had not been clear.**

215: Is this worth reporting, if so explain why.

**Response: We do believe these results are worth reporting. They are part of the same meta-analysis as the life history results. The meta-analysis importantly demonstrates that these isolates have a known history of growth promotion, and that past experience is consistent with these current results. The trends are also consistent with phylosymbiosis, though these trends are not statistically significant. These results reinforce the conclusions from the direct tests of mycorrhizal feedback .**

281: Uniquely robust I believe is true in that many tests have been completed, but see concerns here about meta-analysis power as well as concerns over methods used to test for phylogentic signals (and comparison to species null models).

**Response: There are multiple analyses presented here, each with their own strengths and weaknesses. While the meta-analysis was only able to include 3 studies, these studies used the exact same isolate for each species. This allows for much greater confidence in the conclusions about mutualistic quality. While the meta-analysis approach provides measures of confidence in our conclusion of differential impacts of the fungal isolates on plant family and plant life history categories, it is true that the regression approach to testing feedbacks is looking at the correlation across seven AM fungal species. In that respect there is reduced power compared to our direct tests of feedback. We have now identified this in the text. We also have included a wald test of the overall model with the results QM= 163.3303, df = 34, p-val < .0001. These metrics have been included in the results.**

287: To support this statement it would be helpful to actually test this using the method I suggest in like 142. To compare the variance explained by host phylogeny. Unless this is referring to the meta-analysis?

**Response: Here we are referencing the effect of host plant phylogenetic relationships on the AM fungal community specifically. Our Meta-analysis and PerMANOVA results support our finding. We did include an analysis of phylogenetic heritability in response to the comment on line 142.**

299: I suggest to say "suggest" since these models are not explaining all variance.

**Response: it is true that we are not explaining all the variation. But we do have evidence that both plant life history and plant phylogeny influence the pattern of AM fungal community and genetic composition. We have modified the wording to reflect this result.**

304: Grass plant species? Or those studied here? All plant species feels like a very large claim

**Response: We agree that the pool of plant species that we have sampled is not representative of all plant species. They are representative of plant species from the tallgrass prairie region. We have changed the text to reflect this.**

309-310: See comment for 142

**Response: We have included phylogenetic heritability as supplement S13 and refer to it on line165 of the document.**

318-319: This is assuming host-microbe fitness is aligned, which may not always be the case. See: <https://nam10.safelinks.protection.outlook.com/?url=https%3A%2F%2Fonlinelibrary.wiley.com%2Fdoi%2F10.1111%2Feva.13333&data=05%7C02%7CRob.Ramos%40colorado.edu%7C68a79ed505304fd254ba08ddf9ef7ae7%7C3ded8b1b070d462982e4c0b019f46057%7C1%7C0%7C638941529135455761%7CUnknown%7CTWFpbGZsb3d8eyJFbXB0eU1hcGkiOnRydWUsIlYiOiIwLjAuMDAwMCIsIlAiOiJXaW4zMiIsIkFOIjoiTWFpbCIsIldUIjoyfQ%3D%3D%7C0%7C%7C%7C&sdata=65h5GBvTmFJcl5bIEcmDYVtno1TscqBy657U3nLOYdU%3D&reserved=0>

**Response: It is absolutely true that host and microbe fitness is not inherently aligned. We identify and discuss this in the introduction and refer to examples of fitness asymmetries (See Bever 2002 Proc Roy Soc). In this case we are only referring to greater fitness of AM fungi when associating with plant hosts. We do not draw any conclusions from this result on the fitness of the host plant in the association. We directly test feedback effects in other parts of the experiment.**

323: It is worth saying this is also conducted withing a single plant family, and it is unclear how the dynamics you describe here play out at diff scales, e.g. within species, between orders.

**Response: While we do look at between and within family comparisons using direct tests of feedback. Please see supplement S11 Fig.**

361: What type of system would be the next best test?

**Response: There are many possible directions and we don’t want to suppose which model systems are best. We have our preferences for mycorrhizal systems, and so ectomycorrhizal fungi or rhizobia would be an interesting groups for further exploration. There are numerous model systems however, and we don’t want to suppose that mycorrhizal fungal system is more likely to produce novel insights than say a fig-fig wasp study system.**

415: how many years of divergence?

**Response: The AM fungal species were allowed to differentiate over two growing seasons before being used as inocula in the feedback experiment.**

423: Nex-gen is now here for over 10 years, I think we can just say what type of Illumina sequencing.

**Response: This is a very fair point, and we have changed the wording.**

429: Define the equation please
**Response: It is defined in the methods. We have added a parenthetical to better guide the reader.**

431: With only three studies, what type of statistical power is there to provide confidence in results? Please report the power for the meta-analysis, and mention in the main text. I suspect it is quite low and results should be interpreted with caution.

**Response: We had conducted a wald test of the overall model with the results QM= 163.3303, df = 34, p-val < .0001. These metrics have been included in the results. We note that the statistical power for our test of correlation of plant and fungal relative fitness values (as in Bever 1999 Ecology Letters) is based on 7 AM fungal species, so is based on 7 points.**

461: See above, what type of Illumina sequencing to what depth and where was it conducted?
**Response: We have specified that MiSeq at the University of Kansas was the platform used. Sequencing depth ranged from 1999 to 48273.**

501: I suggest adding in the ICC as mentioned above for these models, to not just give p-values for the phylogenetic relationships but determine magnitude of effect.

**Response: We have added the ICC, i.e. the phylogenetic heritability. We have also added in psuedo R2 values for all pglmm results in response to another reviewer’s comments.**

505: Is Shannon density telling you much here? If this analysis does not add much to your results I suggest removing and simplifying your story.

**Response: Shannon diversity was included as a metric of overall community structure. We wanted to examine the effects of community level changes, species level changes, and within species level changes in this analysis. It is in fact interesting that the Shannon diversity of AMF increases with late successional plant species. It ends up that we don’t have evidence that this drives mycorrhizal feedbacks, but we couldn’t have know that without including and testing this effect.**

506-509: This is a very long sentence that I think should be broken up and explained clearly if this is referring to calculation of growth rates. Please define density. In general I suggest splitting the stats part into subsections based on what you are testing with what data and what tests.

**Response: The explanation for our proxy of density was not as clear as it could have been. We have rewritten that section to try and simplify the sentence structure and clarify the details.**

524: Maybe I am missing it, but what if the fitness measurement, or is it a proxy like biomass?

**Response: The fitness proxy is biomass, as we identify on line 554. These plants were all harvested before any substantial investment in reproduction occurred, and the plants were investing heavily in vegetative growth. Therefore, biomass is a very strong measurement of plant fitness.**

5298: Is this an established method to look at genetic dissimilarity with grouped species from ASVs? Does this speak more to our poor species concept for AM fungi?

**Response: This isn’t a problem of AM fungal species concepts. AM fungi are multinucleate and individual cells of AM fungi are consistently highly polymorphic in the rDNA. Therefore it is always the case that AM fungi have many rDNA ASVs within single species. We introduced this issue in the Introduction.**

Figure captions: Many people only look at figures and captions, can you please write to show the main findings for the figure, what data and tests were used. Great figures stand alone.

**Response: We have attempted to add major findings to figure legends where they were absent throughout.**

Reviewer #2:

Summary:
This study investigates the role of plant phylogeny and life history in structuring the symbiosis between arbuscular mycorrhizal fungi and their herbaceous host plants. In particular, the study takes an experimental glasshouse-based approach in which a common set of 7 AMF species are mixed into soils containing 1 of 38 plant species and they are grown for two seasons. Periodic sampling of the soils is done to estimate AMF relative abundances over the experiment period, which is assessed with high-throughput sequencing. Additionally, a meta-analysis is conducted to estimate the strength of feedbacks for the 7 AMF with some of the hosts used in the experiment based on previous research in this system. The primary research question is whether plant phylogeny, plant life history, or both explain AMF community metrics as well as the strength of plant-AMF feedbacks.

The authors contextualize their study in work in other study systems that have shown that phylogenetic relatedness is an important factor affecting the structure and functioning of symbioses, for which they use the term phylosymbiosis. Given the ecological ubiquity and importance of the AM symbiosis, it is surprising that this has not received more explicit attention, making the relevance of this work high despite the greenhouse setting.

The analyses are structured around testing how change in AMF density and composition is driven by either or both plant phylogeny and life history. This involves applying either phylogenetically explicit GLMs or PERMANOVAs, which give general similar results. The results are also structured at the ASV level, so they allow for assessment of within AMF species variation in performance, which is referred to AMF genotypes. Following this primary analysis, the result then shift to incorporating the results of the meta-analysis to test how the strength of the AMF-plant feedback is impacted by plant phylogeny and life-history. This is primarily done with regression-based analyses.

Given the large number of plants and AMF, it perhaps not too surprising that there is significant variation in the AMF community across the hosts in the different years. The authors find support for effects of both plant phylogeny and life history on AMF community dynamics, but they find limited support for the strength of AMF-plant feedbacks to be clearly structured by phylogeny. There is some significant signal for positive feedbacks with life history, particularly with late successional species, which is consistent with those plants and AMF potentially being more co-dependent. Overall, the results do support some level of importance for both plant phylogeny and life history on AMF community dynamics and on AMF-plant feedbacks, but that there are many genotype, species and clade level patterns that make this relationships complex.

Major comments:

Possibly due the manuscript structure for review, I found the study surprisingly hard to follow. On the plant side, given so much previous work done in this system and possible even with the plants in this experiment (it seems like this may not be the only publication from this experiment?), I wanted to have a clearer explanation in the introduction about the features of this system that make it such a strong test of the question the authors are addressing. Currently, the introduction treats topics at a very generic level targeting any biologist interested in these questions, but given the genotype, species, and clade level patterns observed, I think a more study system specific introduction would be helpful.

**Response: We want to stress the introduction that our work is relevant to multiple audiences and adds significantly to multiple ongoing intellectual conversations in ecology and evolution. You are correct that we do know a lot about the biology of our system and we use that in the current study. We have added a paragraph introducing this background to the introduction.**

**We note that this is the only paper from the current study. By testing across 38 hosts, our work is uniquely robust test of phylogenetic influence on symbiont composition. By integrating this test with a feedback functional assay, our work is one of the few to follow up observations of phylosymbiosis with an explicit test of its functional consequences. By robustly testing symbiont feedback within the most common and ecologically consequential plant symbiont, our work is of broad interest to terrestrial ecologists. By simultaneously testing feedbacks due to ecology (i.e. change in species composition) and evolution (i.e. change in genetic composition within species), our work crosses disciplinary boundaries. We attempted to keep the introduction relatively short while covering the relevant literature to all these important domains.**

On the fungal side, I was also unsatisfied with both the description of the sampling and analyses of the molecular data. For example, how was the soil in the pots sampled, from what depths, and how big were the pots. 0.2 grams of soils is a very small amount, so I could imagine where in the pot the sample was taken from could matter a lot. Regarding the molecular analysis itself, I appreciate the ASV level resolution, although I would like to know the number of ASVs per species were present in the spores to start with. It is very different across the 7 AMF species? The relative abundance analyses are also problematic for comparisons across taxa given the compositional nature of high-throughput sequencing (HTS), so some accounting for compositionality seems required to be sure the results of increases in one taxon are not resulting in compensatory decreases in another taxon just because the total number of sequences is fixed. The authors talk about AMF densities - how is that defined? That should be done with qPCR or some direct measure or quantity, not HTS data. There is little discussion of the fact that spores could be present as sources of DNA by the inoculated species (either residual spores or newly produced spores) so relative abundances reported might be affected by multiple parts of the life cycle of plants. Finally, given all the previous work in this system by these authors, I also wanted to see some discussion of the relationship between soil abundance and root colonization, as both seem important to feedback strength. While these may seem like minor details only important to mycologists, I think without more detailed discussion of the biology of the system itself, it very hard to evaluate the validity of the larger conclusions drawn.

**Response: We have included more detail in the method section on the points raised. I will also address them here. 1) We did take the standard 0.2 grams of soil for sequencing, but soil from each pot was homogenized before sampling. 2) The methods do already specify that the training was done in two-gallon pots and the feedback assay was done in 1-liter pots. 3) We had not specified in the manuscript the number of ASVs per AM fungal species though it was identifiable from Figure 3. We have now explicitly stated that *C. clariodeum* has 4, *C. lamellosum* has 2, *E. infrequens* has 8, *A. Spinosa* has 15, *R. Fulgida* has 9, and *F. mosssea* has 12 ASVs. 4) We mention in the methods that all data in the PerManova is center log transformed. This transformation accounts for compositionality, and as our PerManova results reinforce the results of the PGLMMs we feel confident in the trends we identified. 5) AMF densities were unfortunately identified via proxy with the proportion of AM fungal AVSs over all ASV’s Identified. It is an imperfect proxy, but as we have identified in our new supplement 13, is statistically significantly correlated with spore counts counted in a subset of the samples (p<0.001). 6) It is mentioned in the methods that all soil was steam sterilized. The only living spores in these pots were our inoculated AMF. Those AMF were inoculated in roughly equal proportions at the start of the experiment. We are confident that the changes in relative proportions of ASVs are directly related to changes in fitness of our inoculated AM fungal species. 7) We note that our growth cycles were arranged so that AM fungi were in their dormant structures (i.e. spores) at the end of the growing season. At this point in the AMF life cycle, infection is no longer a meaningful measure of density.**

**We note that this project was a massive amount of work and the analyses that we did were enabled by significant advances in bioinformatics of the LSU region of rDNA. We did not have the funds to simultaneously develop qPCR approaches or score root infection when we did not expect that the data to be useful. In spite of these limitations, we have been able to answer previously unaddressed basic questions on AM fungal biology and feedbacks, and answer questions not previously addressed at this scale in any symbiosis.**

Minor comments:

Some redundancy in the writing - particularly in the discussion. Circling back to take home messages nearly every paragraph is distracting and I suggest the authors try to focus on clean intro and concluding summary paragraphs in the discussion, with the other paragraphs being more specifically targeted at contextualizing the results relative to other similar studies.

**Response: We appreciate this comment, but we chose to actively signpost in the discussion. This was a deliberate choice to better tie our many analyses and approaches to our overall questions. In revisiting the discussion, we have reduced redundancies which the author noted.**

Figure 1 has panels labeled with AMF fitness and Plant Fitness. As far as I can tell, neither number of spores or number of seeds was ever measured in this study. As such, AMF performance and Plant performance seem like more appropriate names.

**Response: Unfortunately, we could not collect a complete dataset for spore counts. The spore counts we were able to collect do statistically significantly correlate with our amplicon proportion. We are testing across plants of very different life histories. Seed count is not necessarily a good proxy of fitness for long lived perennials. Moreover, because we want to test plant response to AMF composition, which we show change over the course of two years in growing with a given plant, using seed production in a perennial is not an option. Our proxy of plant fitness is plant biomass production in a short growth assay. Biomass production in short assays has been shown to be a good proxy for fitness when comparing within plant species and when comparing plants that have not yet allocated to reproduction. That is what we have used. Please see the plant soil feedback literature (Bever et al. 1997 Journal of Ecology and papers that build on that approach) for evidence that this approach has utility. And note that in our metric of feedback, the main effect of plant species (i.e. any difference in biomass between species) cancels out.**

There are small grammatical issues throughout the manuscript that need careful proofing. Examples: infrequens misspelled in a few places, Figure 4 Y axis spelling of response.

**Response: I have addressed the edits, and have also gone back through the manuscript for any additional misspellings.**

Reviewer #3:

The study by Ramos et al. investigates the phylogenetic and plant life history effects on AM fungal species abundance and composition. It is very interesting that they also look at the effects on AM fungal species genetic composition, which is rarely addressed. Overall I find the work compelling and well conducted, but parts of writing needs revising, especially adding more details to Methods sections. I also highlight a few points regarding life history traits of the chosen AM fungal isolates, that might influence preferential hyphal biomass allocation between soil and root compartments, while only soil was sampled.

Line 146-148 This sounds more like Discussion and there are other sentences like that in the results.
**Response: I understand the concern of mixing results and discussion. However, this is a straight forward explanation of the general trend present and is mostly included to help guide the reader in understanding the figure.**

Line 283 It's hard to judge strength of the model just based on p value. Is it possible to provide (marginal) R2-values for the PGLMM models?

**Response: pseudo R2- values for the PGLMM models have been added to supplement 5**

Line 295-296 Check the sentence for clarity.

**Response: The sentence has been edited: "Differential positive feedbacks that vary between plants of different life history characteristics could contribute to plant species turn over during succession."**

I find it curious that out of the 7 AMF taxa used in this study, 2 (R. fulgida and Ce. pellucida) are edaphophilic (making high biomass in soil), 3 are rhizophilic (making high biomass inside roots; C. lamellosum, C. claroideum, F. mosseae) and 2 are ancestral (with no particular preference; E. infrequence, A. spinosa) and both edaphophilic and one ancestral end up being the responsive ones. AMF life history traits are according to Weber et al. 2018. Since soil was sampled and not roots, I'm wondering why only soil was sequenced and not roots and how this affects the conclusions?

**Response: It was not clear in the methods, and we have now clarified soil and roots were homogenized before sampling. Our DNA extractions would therefore include both root and soil DNA. Please note that we mimicked a normal growing season in our greenhouse so that AM fungi produced spores, the dormant structure, which would robust to homogenization and inoculation.**

Overall the Materials and Methods section needs some elaboration and basic details.

**Response: We have made extensive edits to the methods to aid in clarity.**

Please add more details about how the inocula were made, can you be sure that these are single-species isolation, how were they propagated, I guess they were not sterile?

**Response: We have added details into the first paragraph of the methods. “These isolates were inoculated from pure spores into pots of sterilized 50/50 sand soil mix.” We note that we have had these same AM fungal cultures have been maintained as clean cultures in our lab for more than 10 years—grown out new each year. They were used for previous studies as cited in our methods and now with an additonal paragraph in the introduction.**

How were the mesocosms inoculated - how much inoculate (grams or spores?) per isolate per pot?

**Response: The training pots were inoculated from using mixtures of the pure inocula (which contains AMF grown in sterilized soil:sand mix, see INVAM protocols: https://invam.ku.edu/ . Feedback assay pots were inoculated with 100ml of homogenized soil from the training pot.**

What was the potting soil?

**Response: It was a sterilized 50/50 sand soil mix.**

How many pots were there in total?

**Response: There were 152 training pots (4 replicates of each of 38 species) grown in each of two growing seasons, and 1,464 feedback assay pots.**

Line 410-413 What was the selection criteria for choosing these particular AM isolates?

**Response: We chose these isolates because we had them in culture from native prairie remnants and they had previously been used in many growth assays, so we had a lot of data on how these AM fungi impacted prairie plant species, which we use in our metadata analysis and tests with these results.**

Line 420 use SI units instead of gallon

**Response: We generally use SI units where appropriate. However, in this case these pots are standard two-gallon pots that are sold in many greenhouse supply catalogs. They are sold under that term as well, and so it might be confusing to refer to them in SI units.**

What is over-representation and under-representation of sequences? Compared to what?

**Response: Overrepresentation (as proportion of total sequences or CLR transformed counts) when compared to the other pots in the experiment.**

Line 426 not sure the word "trained" captures the meaning, perhaps "conditioned" is better?

**Response: We have changed our terminology to conditioned.**

Line 427 what other plants?

**Response: This was unnecessarily confusing. We have specified that we are referring to the same 38 plant species used in the conditioning step.**

Line 428 Liter does not constitute depth

**Response: Deep pots are the type of pots sold by our particular greenhouse catalog. It is probably too general of a term to be helpful, so we have removed that wording to prevent confusion.**

Line 444 add functions and versions for all mentioned packages

**Response: Individual function names for the standard functions in a packages seems like it would be overly specific. It is something that can be gleamed from package documentation and would only add clutter to the methods. We have added version names to the software used.**

Line 446 phylogenetic instead of phylogenic

**Response: Thank you, we have made the correction.**

Line 452-454 citation style not complying with journal standards

**Response: We have updated those citations to follow the numbered style.**

Bioinformatics Methods - first paragraph is about molecular methods, consider making a separate paragraph on Molecular Methods. Please revise for clarity- E.g line 461 "Extraction was performed…" Extraction of what? PCR conditions are described, but could you please specify how is this related to Illumina sequencing? These do not seem to contain tagged primers? What about Illumina chip that was used? 2x250 or 2x300bp?

**Response: Additional details have been provided in the text.**

Line 478- 481 What was the proportion of non-target sequences? It could also be assumed that legacy DNA might be rather degraded after sterilization (which method was used?), thus not really amplifying long reads. Could the non-targeted DNA also originate from isolates, thus not having single-species but sometimes mixed-species isolates due to contamination issues etc? Sequencing isolates themselves would verify this. Also, did you sequence plant roots - this would also tell you if the non-targeted DNA was from live AMF or not? If you had control pots with only sterile pots, it would be possible to track contamination from spore dispersal through air etc.

**Response: Our proxy for density, though limited, is the proportion of AM fungal ASVs to the sequencing depth. This ranged from 0.01 - 0.20. Contamination is always a top concern. These cultures are prepared with extreme care and there is no evidence that they were not pure single species AM fungal cultures at the time of inoculation. In fact, we continue to maintain these cultures and know that there is no contamination. The non-target DNA then is from the autoclaved soil:sand mix that is the background for all our cultures and experiments. Soil and roots were homogenized before sampling. Our DNA extractions would therefore include both root and soil DNA. We did have sterile pots as that information is crucial for determining log response ratios.**

Were the PCR product concentrations equalized before sequencing? How would that influence read abundances?

**Response: They were, and this has now been clarified in the methods.**

Was the sequencing data standardized? How much did sequencing depth vary between samples?

**Response: We rarified sequences before calculating Diversity metrics. For our multivariate analysis, we also used the CLR transformation which does not require standardization before the transformation is applied. PGLMM results were logit transformed and not standardized beforehand (except for diversity), but as they were fully consistent with our multivariate approach, we feel confident in the results.**
