# Supplementary material for: Host plant phylogeny predicts arbuscular mycorrhizal fungal communities, but plant life history and fungal genetic change predict feedback
Source: PLoS Biol. 2026 Feb 25;24(2):e3003304. doi: 10.1371/journal.pbio.3003304 (PMC12962545; doi:10.1371/journal.pbio.3003304)
Supplement: Attachment — Submitted filename: response_to_reviewers_2026-01-28.docx [file pbio.3003304.s016.docx]

**Reviewer #1:**

The authors have done considerable work to improve the readability of the manuscript and support the large amount of experimental work and analyses. I have a few minor comments.

**Response: We appreciate the very constructive feedback, and have attempted to address all the remaining concerns.**

AM fungal density: I am not fully persuaded that this is a reliable proxy. If this metric is commonly used, please add a citation. If not, stronger justification is needed. Related to this, I could not find a clear description of the model used for Fig. S13 in the Methods or in the figure caption. The fitted line in Fig. S13 has very narrow uncertainty, whereas the scatter of observed points looks very weak. If the relationship between spore counts and fungal density is weak, then confidence in density as a proxy should be low. Please revisit Fig. S13, provide the model details (name, variance explained, and clarify the definition and rationale of the density metric in the Methods (around line 535).

**Response: We have added the McFadden pseudo R2 (0.051) into the figure legend for S8 Fig (formerly S13). We have also specified that the blue line is the best fit line using a Poisson generalized linear model. We also added the following text to the manuscript: “The low McFadden pseudo R2 (0.051) however shows that this is a weak proxy. We include it in the final analysis as a best estimate and note that our overall conclusions remain consistent with our without the consideration of fungal density.”**

Figures: Several figure legends would benefit from more depth in explanation of the figures. For example, in Figure 5 it is not clear what the grey line represents. Please specify whether this is a fitted model, mean trend, or other summary and screen all figures for clarity. Please check spelling in Fig S9. In Supplemental be consistent with figure naming, some are title case others are not.

**Response: We have gone through and made sure the figure names are now consistent. We have also specified in the figure legend that the line in Figure 5 is the regression line.**

Fig S9. 99.999% seems like a very unique number, can this be confirmed? Is this a property of the model, since it is essentially 100% of variance explained it feels very high.

**Response: These are based on the ratio of residual variation explained by phylogenetic relationships to the variation explained by both phylogenetic relationships and species to species variation. If there is very little variation explained by the individual species effect, you can get near 100% values.**

Methods: Please report greenhouse growing conditions, including temperature and light.

**Response: We have added additional details in the methods. “Initial soil training was conducted in a climate controlled greenhouse. Temperatures ranged from 70-90 F, a broad spectrum light system was used 14 hours a day. The greenhouse had a closed HVAC system and humidity was kept at approximately 50%. Pots had drip irrigation with approximately 70 ml of water administered daily… The feedback experiment was conducted in a greenhouse cooled with a swamp cooler, exposed to ambient lighting conditions, and similarly drip irrigated.”**

Please explain how model fits were assessed (e.g. man

Line ~533: "Hints" is not sufficient for a scientific manuscript, rewrite.

**Response: This line was removed.**

Lines ~550-565: Many transformations to data have been conducted in this section (log, logit) and is unclear if this was necessary. Such transformations of data should only be done if there is concern over violating model assumptions. Suggest saying assumptions were tested (e.g. residual normality) and then including transformations only if necessary.

**Response: Logit transformation is a common transformation for community and population level analysis. We did test for normality and found that the logit transformation was appropriate. That analysis is provided in the code for the figures and analysis.**

Line 577: Is block the greenhouse block? Likewise "log transformed sequences" are the DNA sequences? Or many I am not following, please clarify.

**Response: We have specified in the text: “Greenhouse block and the log transformed AM fungal dna sequences were included alongside phylogenetic distance and species as random effects.”**

**Reviewer #3:**

I feel that the authors have done a great job making the manuscript clearer, especially the methods section. I am satisfied with their responses to my comments (I was reviewer no 3) and have no further issues.
